# Real-world usage of Chronic Kidney Disease – Mineral Bone Disorder (CKD–MBD) biomarkers in nephrology practices

**DOI:** 10.1093/ckj/sfad290

**Published:** 2023-11-29

**Authors:** Maria Fusaro, Simona Barbuto, Maurizio Gallieni, Althea Cossettini, Giulia Vanessa Re Sartò, Laura Cosmai, Giuseppe Cianciolo, Gaetano La Manna, Thomas Nickolas, Serge Ferrari, Jordi Bover, Mathias Haarhaus, Carmela Marino, Maria Cristina Mereu, Maura Ravera, Mario Plebani, Martina Zaninotto, Mario Cozzolino, Stefano Bianchi, Piergiorgio Messa, Mariacristina Gregorini, Lorenzo Gasperoni, Caterina Agosto, Andrea Aghi, Giovanni Tripepi

**Affiliations:** National Research Council (CNR), Institute of Clinical Physiology (IFC), Pisa, Italy; Department of Medicine, University of Padova, Padova, Italy; Nephrology, Dialysis and Renal Transplant Unit, IRCCS-Azienda Ospedaliero-Universitaria di Bologna, Alma Mater Studiorum University of Bologna, Bologna, Italy; Department of Biomedical and Clinical Sciences ‘Luigi Sacco’, Università di Milano, Milano, Italy; Post-Graduate School of Specialization in Nephrology, University of Milano, Milano, Italy; Division of Nephrology and Dialysis, Azienda Socio-Sanitaria Territoriale (ASST) Fatebenefratelli-Sacco, Fatebenefratelli Hospital, Milan, Italy; Post-Graduate School of Specialization in Nephrology, University of Milano, Milano, Italy; Post-Graduate School of Specialization in Nephrology, University of Milano, Milano, Italy; Division of Nephrology and Dialysis, Azienda Socio-Sanitaria Territoriale (ASST) Fatebenefratelli-Sacco, Fatebenefratelli Hospital, Milan, Italy; Nephrology, Dialysis and Renal Transplant Unit, IRCCS-Azienda Ospedaliero-Universitaria di Bologna, Alma Mater Studiorum University of Bologna, Bologna, Italy; Nephrology, Dialysis and Renal Transplant Unit, IRCCS-Azienda Ospedaliero-Universitaria di Bologna, Alma Mater Studiorum University of Bologna, Bologna, Italy; Department of Medicine, Division of Nephrology, Columbia University, New York, NY, USA; Service des Maladies Osseuses, Département de Médecine, HUG, Geneva, Switzerland; Servicio de Nefrología, Hospital Universitario Germans Trias i Pujol, Badalona (Barcelona), Spain; Division of Renal Medicine, Department of Clinical Science, Intervention and Technology, Karolinska University Hospital, Karolinska Institutet, Stockholm, Sweden; National Research Council (CNR), Institute of Clinical Physiology (IFC), Reggio Calabria, Italy; Independent Researcher, Cagliari, Italy; Nephrology, Dialysis, and Transplantation, University of Genoa and Policlinico San Martino, 16132 Genoa, Italy; Laboratory Medicine Unit, Department of Medicine, University of Padua, Padua, Italy; Laboratory Medicine Unit, Department of Medicine, University of Padua, Padua, Italy; Department of Health Sciences, Renal Division, University of Milan, ASST Santi Paolo e Carlo, Milan, Italy; Department of Internal Medicine, Nephrology and Dialysis Complex Operative Unit, Livorno, Italy; Nephrology, Dialysis and Renal Transplantation, Fondazione IRCCS Ca' Granda Ospedale Maggiore Policlinico, Milan, Italy; SC Nefrologia e Dialisi AUSL-IRCCS Reggio Emilia, Reggio Emilia, Italy; Nephrology and Dialysis Unit, Infermi Hospital, AUSL Romagna, Rimini, Italy; Pediatric Pain and Palliative Care Service, Department of Women's and Children's Health, Padua University Hospital, Padua, Italy; Independent Researcher, Padua, Italy; National Research Council (CNR), Institute of Clinical Physiology (IFC), Reggio Calabria, Italy

**Keywords:** bone biomarkers, bone fractures, bone turnover markers, CKD-MBD, vascular calcifications

## Abstract

**Background:**

Chronic kidney disease mineral bone disorder (CKD-MBD) is a condition characterized by alterations of calcium, phosphate, parathyroid hormone (PTH), and fibroblast growth factor 23 (FGF-23) metabolism that in turn promote bone disorders, vascular calcifications, and increase cardiovascular (CV) risk. Nephrologists’ awareness of diagnostic, prognostic, and therapeutic tools to manage CKD-MBD plays a primary role in adequately preventing and managing this condition in clinical practice.

**Methods:**

A national survey (composed of 15 closed questions) was launched to inquire about the use of bone biomarkers in the management of CKD-MBD patients by nephrologists and to gain knowledge about the implementation of guideline recommendations in clinical practice.

**Results:**

One hundred and six Italian nephrologists participated in the survey for an overall response rate of about 10%. Nephrologists indicated that the laboratories of their hospitals were able to satisfy request of ionized calcium levels, 105 (99.1%) of both PTH and alkaline phosphatase (ALP), 100 (94.3%) of 25(OH)D, and 61 (57.5%) of 1.25(OH)_2_D; while most laboratories did not support the requests of biomarkers such as FGF-23 (intact: 88.7% and c-terminal: 93.4%), Klotho (95.3%; soluble form: 97.2%), tartrate-resistant acid phosphatase 5b (TRAP-5b) (92.5%), C-terminal telopeptide (CTX) (71.7%), and pro-collagen type 1 N-terminal pro-peptide (P1NP) (88.7%). As interesting data regarding Italian nephrologists’ behavior to start treatment of secondary hyperparathyroidism (sHPT), the majority of clinicians used KDOQI guidelines (*n* = 55, 51.9%). In contrast, only 40 nephrologists (37.7%) relied on KDIGO guidelines, which recommended referring to values of PTH between two and nine times the upper limit of the normal range.

**Conclusion:**

Results point out a marked heterogeneity in the management of CKD-MBD by clinicians as well as a suboptimal implementation of guidelines in Italian clinical practice.

## Graphical Abstract

**Graphical Abstract:**
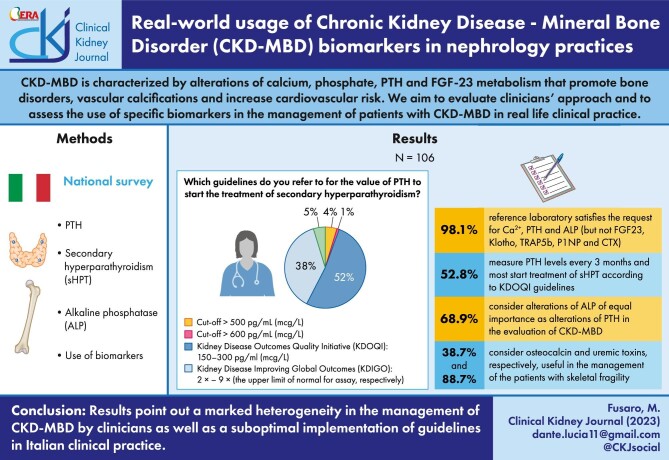

## INTRODUCTION

Progression of renal damage is directly associated with the genesis and development of hyperparathyroidism, with recent evidence pointing to a fundamental role of the Klotho-FGF-23-FGF receptor (FGFR) axis. The onset of CKD, already in the early stages, is associated with a decrease in the number of functional nephrons with a consequent reduction in phosphate excretion per nephron, then the increase in FGF-23 (bone-derived hormone) and PTH are necessary to maintain phosphate balance by exerting a tubular phosphaturic effect. This occurs through the binding between FGF-23 and the αKlotho-FGFR receptor complex; αKlotho is a transmembrane protein expressed in many tissues and especially in the kidney distal convoluted tubule and in the parathyroid chief cells [1]. Another effect of the increase of FGF-23, mainly related to phosphate retention, is the reduction of expression and activity of 1α-hydroxylase in renal tubular cells, leading to a decreased production of 1.25(OH)_2_D (calcitriol) from 25(OH)D [2]. The lower calcitriol action on the vitamin D receptor (VDR) induces a reduction in intestinal calcium absorption and a decrease in serum calcium, which stimulates the synthesis and release of PTH. Furthermore, the increase in PTH stimulates the rise in FGF-23 by acting on osteocytes/osteoblasts and increasing calcitriol, and, vice-versa, FGF-23 suppresses the secretion of PTH by acting on parathyroid cells again through the FGF-23-αKlotho-FGFR receptor complex. These compensatory mechanisms seek to normalize serum calcium by stimulating 1α-hydroxylase and increasing bone turnover and resorption, as well as seeking to reduce serum phosphate with the phosphaturic action of PTH, added to that of FGF-23 [3]. The persistence of abnormal calcium and phosphate metabolism and the reduction of bone response to PTH makes this compensation ineffective and promotes the progression of secondary hyperparathyroidism (sHPT) [4].

Bone involvement in CKD-MBD is referred to as renal osteodystrophy, characterized by histological bone changes that include abnormalities in turnover (high/low), mineralization, and bone volume, leading to abnormal cortical bone structure and quality, which negatively affect bone strength [5]; in this context, diagnosis and management are complex [6]. Patients with advanced CKD have a narrow cortical width, which is likely associated with reduced bone strength. Cortical thinning, which is generally irreversible in dialysis patients, is due to severe bone resorption of the endocortical surface and impaired periosteal apposition [7].

It should be noted that the role of osteocytes in bone mineral homeostasis, in particular osteocytic osteolysis [8], is underestimated in guidelines and probably ignored by most clinicians. Over 90% of bone cells are osteocytes. Osteoclastic bone resorption accounts for only 0.1% of total calcium release in non-CKD subjects, which indicates that osteocytic osteolysis is very important for efficient calcium homeostasis. The role of osteocytes in bone mineralization, their influence in the control of bone resorption by osteoclasts and bone formation by osteoblasts could in the future give important clues to clinicians for the prevention and management of CKD-MBD. In fact, the receptors of vitamin D [9], estrogen [10], and PTH [11] were found on the osteocyte, supporting the concept that the osteocyte significantly affects mineral homeostasis.

In particular, in CKD-MBD a reduction in the number of osteocytes and an abnormal mineralization by the remaining ones occur, causing extensive hypomineralization of the bone that increases its fragility and leads to an increased risk of fracture. Proper bone mineralization by the osteocyte population is crucial to increase bone mineral density (BMD). It was observed that, in a population with renal hyperparathyroidism treated with parathyroidectomy and with a moderate concomitant intake of active vitamin D (alphacalcidiol), the mineralization of osteocytes was greater than that of osteoblasts. However, it was observed that, in the absence of a correct intake of vitamin D, the area of hypomineralization was higher, highlighting the pivotal role of vitamin D in ensuring proper bone mineralization by osteocytes [12].

Bone densitometry (DXA) of the hip and spine, useful for evaluating fracture risk in the general population, fails to adequately discriminate bone quality (evaluating cortical and trabecular microarchitecture); moreover, it is not diagnostic for alterations in mineralization and bone volume [13]. Instead, it is essential to define bone quality because both microarchitectural components (cortical and trabecular) tend to decrease in CKD-MBD, exponentially increasing the risk of bone fragility and fractures. The bone biopsy would represent the gold standard for the evaluation of renal osteodystrophy but, unfortunately, it is rarely performed due to the lack of technical, clinical, and pathological skills, as well as the high costs [14].

Over the years, reliable and inexpensive tests with adequate sensitivity and specificity have been developed to evaluate markers of bone formation or resorption, allowing a non-invasive approach to evaluate high and low turnover [15]. From a strictly pathophysiological point of view, the term bone turnover marker (BTM) should be reserved for a molecule generated within the bone tissue in the process of bone turnover and mineralization. Therefore, although PTH represents a crucial driver of bone metabolism and it is the most tested molecule in clinical practice to define the underlying bone turnover, it should be considered a biomarker and not a BTM [6]. Furthermore, it is important to consider some pitfalls hidden in measurement of PTH, which depend both on the molecule itself and on the measurement method. It is necessary to pay attention to pre-analytical variability considering the influence of patient characteristics (sex, age, ethnicity, BMI, and dietary calcium intake), sampling site (in patients on dialysis arteriovenous fistula or central venous catheter), and the circadian and seasonal variations of PTH. Moreover, there are three generations of assays for dosing PTH [16]. The first-generation kits, due to their low accuracy, have been discarded; currently both second and third generation kits are in use. For establishing an adequate diagnosis, especially in the context of CKD, the type of assay must be considered, as both use the immunoradiometric method. The second-generation kits (intact PTH) reduce the interference from the C-terminal portion and fragments, but they probably have a cross-reactivity with the 7–84 PTH fragment. The consequent overestimation of hyperparathyroidism may influence therapeutic choices. The third-generation kits (whole or bio intact PTH) instead do not read the 7–84 PTH fragments and for this reason they should better define bone turnover alterations in patients with CKD. Few studies to compare the two kits have been performed [17]. Fundamentally, however, it must be considered that although the KDIGO and KDOQI guidelines provide reference ranges for PTH, there is a lack of standardization of the PTH assay. Different strategies have been considered to solve the problem; the most accepted is the proposal by Souberbielle *et al*. of a correction factor, which allows for the comparison of the different assays [17].

Procollagen type1 amino-terminal propeptide (P1NP), osteocalcin (OC), and bone alkaline phosphatase (bALP), among BTMs, are expressions of bone formation. In particular, bALP is an isoenzyme that expresses osteoblastic activity and has four different isoforms (B/I, B1, B2, and B1x, which is present only in patients with CKD). Several studies have shown that bALP levels have high specificity and sensitivity in predicting low and high bone turnover. In particular, its association with PTH values is extremely useful in assessing bone turnover [18]. Carboxy-terminal cross-linked telopeptides of type 1 collagen (CTX) and tartrate-resistant acid phosphatase-isoform 5b (TRAP-5b) are indicators of bone resorption [15]. To avoid bias related to renal retention, BTMs that are not cleared by the kidneys, such as bALP, P1NP, and TRAP-5b, should be considered in the setting of CKD [13]. BTMs are summarized in Table 1.

**Table 1:** Bone biomarkers in clinical assessment.

Biomarker	Role of the biomarker	Association with turnover type	Renal clearance [47]	Age (a), gender (g), menopause (m)
PTH	Regulator of bone metabolism. PTH has high biological variability and test standardization problems as limitations. In addition, reduced skeletal sensitivity (hyporesponsiveness) may occur in patients with CKD.	Despite limitations, iPTH is able to discriminate high bone turnover with a similar accuracy (90% positive predictive value) to that of other biomarkers [31].	No	a,g,m = no
Bone-specific alkaline phosphatase (bALP)	Enzyme expressed in osteoblasts. It hydrolyzes the inorganic pyrophosphate (PPi) which is a mineralization inhibitor. Marker of bone formation [15, 48].	High and low values are associated, respectively, with high and low turnover [31, 32].	No	a,g,m = yes
Osteocalcin	It is the main non-collagen Gla protein of bone, it influences the mineralization of osteoids binding to hydroxyapatite [15]. Marker of bone formation [15, 48].	Low values may indicate low turnover and adynamic bone disease [49].	Yes	a,g,m = yes
Intact-Procollagen type 1 N-terminal propeptide (Intact P1NP or trimeric P1NP)	Fragment released when type 1 collagen is deposited in the bone matrix during bone formation process [32]. Marker of bone formation [15, 48].	High and low values are associated respectively with high and low turnover [31, 32].	No	a,g,m = yes
Procollagen type 1 C-terminal propeptide (P1CP)	Fragment released when type 1 collagen is deposited in the bone matrix during bone formation process [32]. Marker of bone formation [15, 48].	Due to its short half-life, it is more appropriate to use P1NP [47].	Yes	a,g,m = yes
Carboxy-terminal cross-linking telopeptide of type 1 collagen (CTX) Amino-terminal cross-linking telopeptide of type 1 collagen (NTX)	Fragments cleaved from type 1 collagen by cathepsin-K during bone resorption [15]. Markers of bone resorption [31].	Predictive of high bone turnover [31].	Yes	a,g,m = yes
Tartrate-resistant acid phosphatase isoform 5b (TRAP-5b)	Isoform of acid phosphatase isoform. It is found in osteoclasts and cuts type 1 collagen into fragments. Marker of bone resorption [31].	High and low values are associated, respectively, with high and low turnover [31, 32].	No	a,g = yes m = no
Sclerostin	Regulator of bone metabolism. Is an inhibitor of the (Wnt)/β-catenin signaling pathway and thus inhibits bone formation. It reduces osteoblastogenesis and induces osteoclastogenesis [47].	Positive predictor of high turnover and number of osteoblasts [47].	Yes	a,g,m = no

A further critical issue of CKD-MBD is vascular calcifications, leading to an increased risk of cardiovascular events and death [18]. The importance of assessing vascular calcifications is emphasized by the European Consensus Statement on the diagnosis and management of osteoporosis in CKD stages G4–G5D, which suggests evaluating their presence in the aorta when imaging for vertebral fracture evaluation is performed [13]. The observational, multicenter, cross-sectional EVERFRACT study showed that vascular calcifications were higher in patients on dialysis compared to patients with primary osteoporosis and normal kidney function. Aortic calcifications were strongly associated with low values of 25(OH)D, an increase in calcium values, and vertebral fractures. It is also important to note that patients with aortic and iliac calcifications had median OC levels lower than controls (164 vs 288 mg/L, *P* < .001) [19]. Similar results were obtained in the VIKI Study: interestingly, iliac calcifications were associated with vertebral fractures, lower osteocalcin, and lower MK7 (a vitamer of vitamin K) levels [20].

Moreover, the KDIGO guidelines suggest in patients with CKD G3a—G5D to practice a lateral abdominal radiograph to evaluate the presence of vascular calcifications and an echocardiogram for assessing valve calcifications [21]. The Kauppila and Adragao scores are useful for evaluating the presence of abdominal and iliac calcifications on radiography, but they are semiquantitative and poorly reproducible [22]. Fusaro *et al*. recently proposed a novel continuum score based on quantitative computer-assisted tracking of calcifications that can improve the detection and follow-up of vascular calcifications even in the short term, which is not possible with the Kauppila score [23].

The management of CKD-MBD diverges among clinicians notwithstanding guidelines. Indeed, the utility of serum BTMs is still controversial during the treatment of secondary hyperparathyroidism in dialysis patients because of the different impact of the treatments on periosteal, intracortical, endosteal, and cancellous bone surfaces [24]. The purpose of this survey is to evaluate the clinicians’ general approach and to assess the use of specific biomarkers in the management of patients with CKD-MBD in real-life clinical practice.

## MATERIALS AND METHODS

The survey consisted of a multiphase project: (i) identification of a questions set related to the topic being investigated; (ii) launch of the survey within a web platform; (iii) data analysis and synthesis and (iv) data interpretation and drafting of manuscript.

A set of 15 closed questions (Table 2) was formulated regarding the following topics: laboratory tests in clinical practice, use of PTH and management of sHPT, use of alkaline phosphatase (ALP), use of other biomarkers, and utility of osteocalcin and uremic toxins in the management of skeletal fragility.

**Table 2:** Questions included in the survey. All questions were closed, for an easier analysis.

Set of questions	Question	Possible answers
1: **Availability of markers from reference laboratory**	1. Can the reference laboratory of your center fully satisfy all biomarkers requests that patients need in relation to diagnosis and monitoring of CKD-MBD?	Yes or no for the following markers: *ionized calcium, PTH, ALP, 25(OH)D and 1.25(OH)_2_D, fibroblast growth factor-23 intact (iFGF-23) and c-terminal fragments (cFGF-23), klotho (soluble), vitamin K, OC, matrix Gla protein (MGP), P1NP, CTX, TRAP-5b.*
2: **Use of PTH and management of secondary hyperparathyroidism (sHPT)**	2. In my center I rate PTH:	Every other month
		Monthly
		Every 3 months
		Every 6 months
	3. Which method is currently used in your reference laboratory to determinate PTH?	2nd generation method
		3rd generation method
		I don't know
	4. If patient has both high levels of PTH and phosphate, what alteration do you treat first?	Phosphate
		PTH
		I consider them both equally important and I treat them at the same time
	5. Related to the parameters used to define the presence of sHPT, how many patients (%) in CKD 4–5D stage have this metabolic alteration?	>50%
		Between 10–20%
		Between 20–30%
		Between 30–40%
		Between 40–50%
	6. Which guidelines do you refer to for the value of PTH to start the treatment of sHPT?	Cut-off >500 pg/mL (mcg/L)
		Cut-off >600 pg/mL (mcg/L)
		Kidney Disease Outcomes Quality Initiative (KDOQI): 150–300 pg/ml (mcg/L)
		Kidney Disease Improving Global Outcomes (KDIGO): 2$ \times -$9$ \times $ the upper limit of normal for assay
		None of the above
3: **ALP use**	7. In my center I rate ALP:	Every other month
		Annually
		Monthly
		Every 3 months
		Every 6 months
		Occasionally, if indicated by the values of calcium, phosphate, and PTH
	8. During the evaluation of CKD-MBD, how do you consider ALP as a predictor of fracture event?	Of equal importance as PTH
		More important than PTH
		I don't consider it
4: **Other biomarkers use**	9. In my center I rate 25(OH)D:	Every other month
		Annually
		Every 3 months
		Every 6 months
		According to the values of calcium, phosphate and PTH
	10. When do you consider the determination of FGF-23 and Klotho in patients with CKD-MBD?	Never
		Only in some cases
		Always to monitor the patient
	11. If the determination is performed in your laboratory, do you refer to P1NP in your patients?	Up to stage CKD3A
		Up to stage CKD3B
		Up to stage CKD4-5D
		In every stage
		Never
	12. If the determination is performed in your laboratory, do you refer to CTX in your patients?	Up to stage CKD3A
		Up to stage CKD3B
		Up to stage CKD4-5D
		In every stage
		Never
	13. If the determination is performed in your laboratory, do you refer to TRAP-5b in your patients?	Up to stage CKD3A
		Up to stage CKD3B
		Up to stage CKD4-5D
		In every stage
		Never
5: **Osteocalcin and uremic toxins in skeletal fragility**	14. If the determination is performed in your laboratory, do you consider total and/or decarboxylated OC a biomarker of clinical utility in skeletal fragility?	No
		Yes
		Only in some cases
	15. In your opinion, in patients undergoing dialysis treatment, could the determination of uremic toxins play a role in the reduction of skeletal fragility?	No
		Yes

All questions were thoroughly discussed within the Organizing Committee of a series of webinars aimed at achieving a comprehensive but quick and easy-to-answer questionnaire. A web platform (Google Forms) was used to send and collect the answers; the survey was repeatedly sent out to all members of the Italian Society of Nephrology (SIN, Società Italiana Nefrologia) from July 2021 to September 2021. A letter of presentation to explain the aims and purposes, as well as instructions on how to fill the form, was provided as an introduction to the survey. At the end of collection, data were exported to an Excel file and analysed through STATA software. Results were expressed as absolute frequencies and rates and the confidence interval (95%) was calculated using Mid-P exact method.

The biomarkers considered in the survey are all carried out using immunochemiluminescent methods applied to the most common automatized platforms available in the clinical laboratory setting, with the exception of sclerostin evaluated using manual ELISA assays. The serum and lithium-heparin plasma are the most common type of matrix adopted by laboratories according to the manufacturer's specifications. The analytical performance for all tests is routinely monitored using the quality control materials (IQC) and participating to external quality assurance (EQA) schemes, the recommended procedure adopted by all clinical laboratories for the monitoring of quality performance of all tests in the routine setting.

## RESULTS

From July to September 2021, a total of 106 nephrologists participated in the survey for an overall response rate of about 10% of the target population, i.e. the members of the Italian Society of Nephrology. We evaluated two important topics: how nephrologists use PTH and BTMs in clinical practice and how they refer to current guidelines in this regard.

### Use of PTH and BTMs in clinical practice

**Figure 1:**
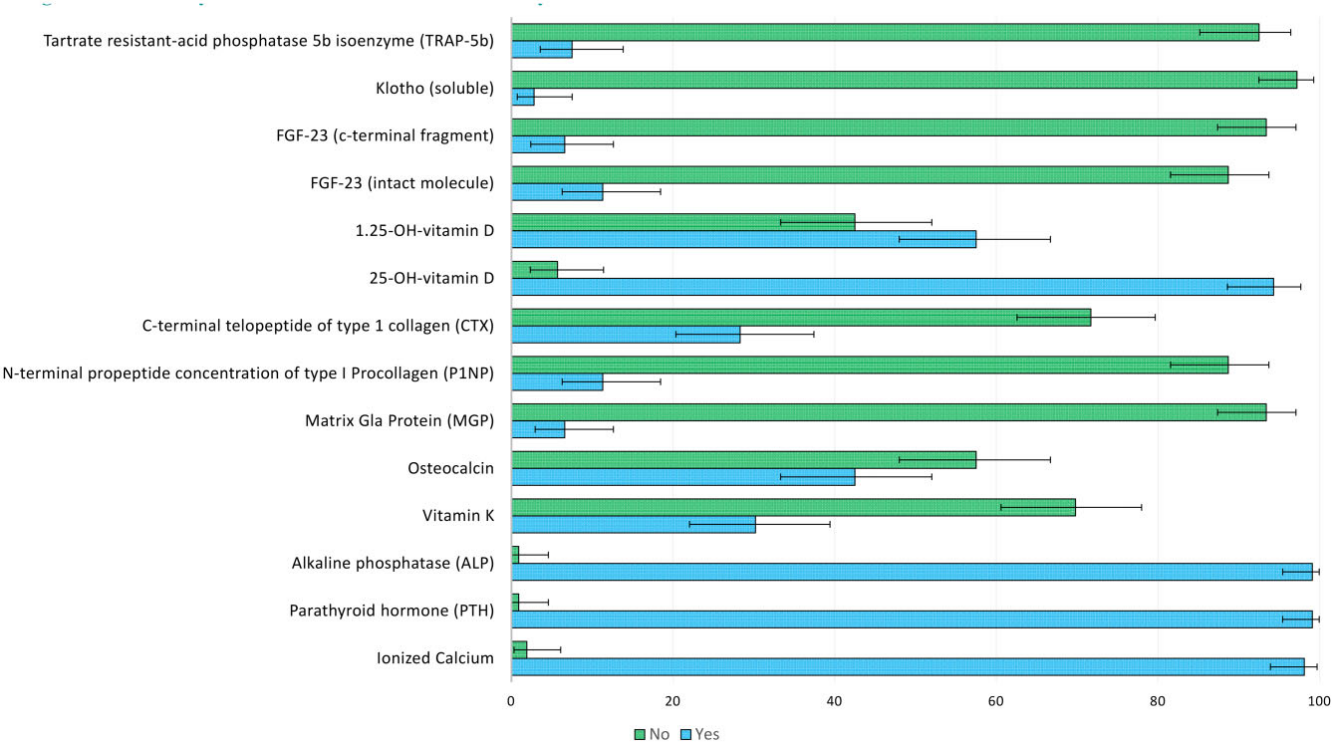
Availability of markers from reference laboratory.

Regarding the first question pertaining to the availability of reference laboratories to satisfy the requests of measuring specific biomarkers, 104 nephrologists out of 106 (98.1%) indicated that the laboratories of their hospitals were able to satisfy the request of ionized calcium levels, 105 (99.1%) of both PTH and ALP, 100 (94.3%) of 25(OH)D, and 61 (57.5%) of 1.25(OH)_2_D. Moreover, most laboratories did not support the requests of biomarkers such as FGF-23 (intact: 88.7% and c-terminal: 93.4%), Klotho (95.3%; soluble form: 97.2%), TRAP-5b (92.5%), CTX (71.7%), and P1NP (88.7%). As for the measurement of OC and vitamin K, the possibility offered by the reference laboratories to provide the assessment of such biomarkers ranged from 30.2% to 42.5%; few laboratories were able to measure Matrix Gla Protein (*n* = 7, 6.6%) (Fig. 1; Table S1, see online supplementary material). Overall, 41 (38.7%) and 26 (24.5%) physicians indicated the use of the second and third-generation kit for PTH measurement, respectively, whereas 39 participants (36.8%) did not know the kit used for measuring PTH in their center.

**Figure 2:**
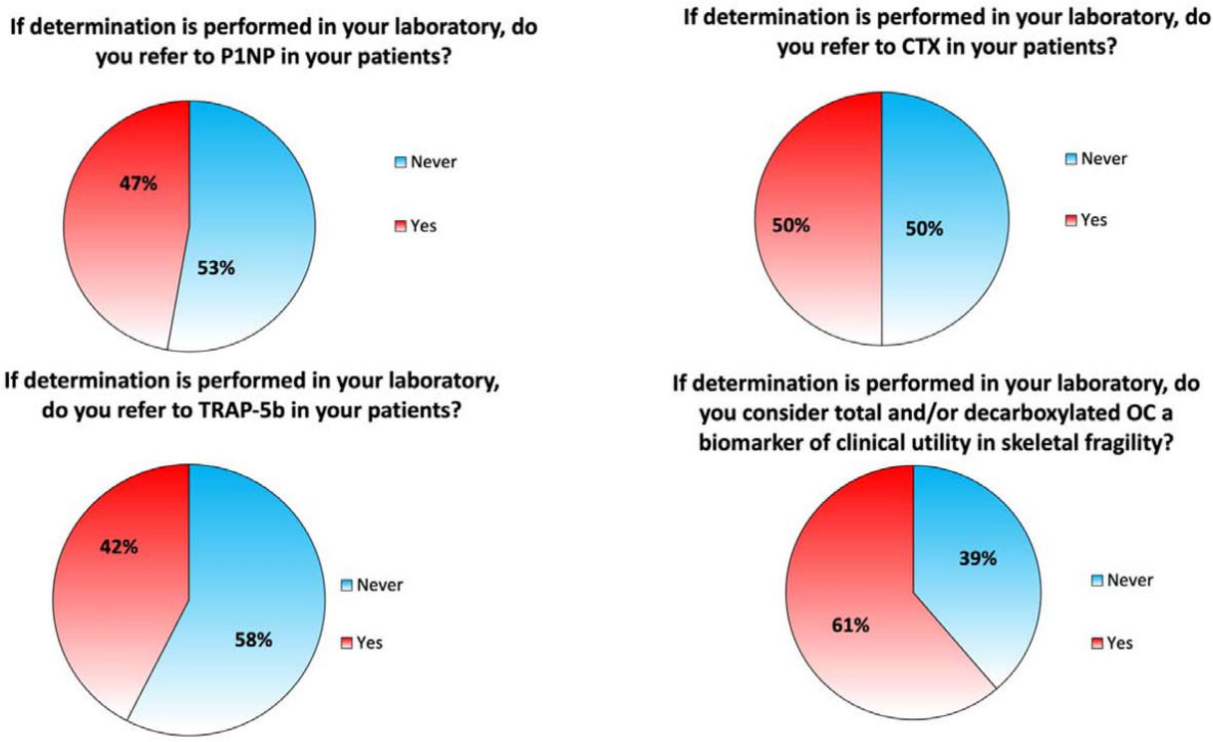
Use of other biomarkers.

**Figure 3:**
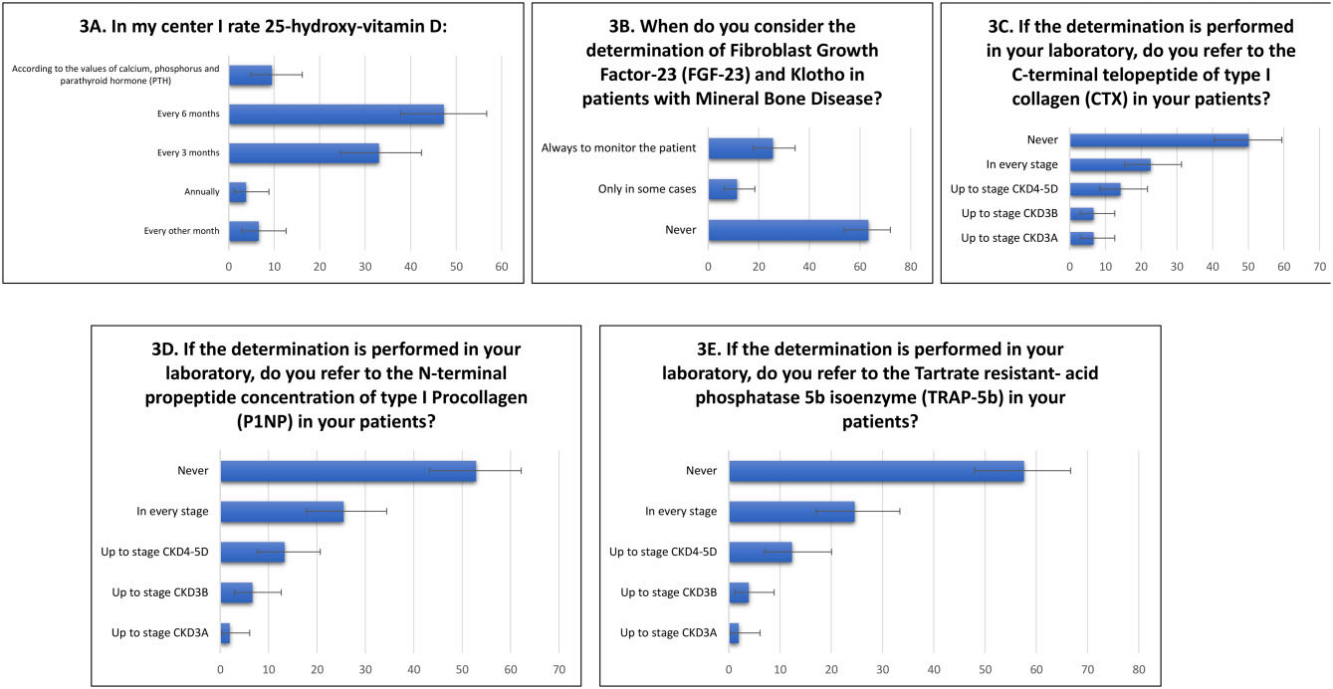
Ratings of 25(OH)vitaminD, FGF-23, Klotho, CTX, P1NP, and TRAP-5b in detail.

Bone turnover markers in clinical practice are rarely tested (Figs 2**–**3; Table S2, see online supplementary material). In fact, apart from measurement of 25(OH)D levels, required by 50/106 participants (47.2%) every 6 months, 35 (33%) every 3 months and 10 (9.4%) according to the values of calcium, phosphate, and PTH (Fig. 3A); only 27 clinicians (25.5%) consider determination of FGF-23 and Klotho to monitor the patients with CKD-MBD, while 67 clinicians (63.2%) never consider them (Fig. 3B). P1NP is never requested by most nephrologists (*n* = 56, 52.8%) and only 14 (13.2%) require this biomarker in patients with CKD4-5D (Fig. 3C). Similar results were found for CTX and TRAP-5b which are never requested by 53 (50%) and 61 (57.5%) participants and limited to patients with CKD4-5D by 15 (14.2%) and 13 (12.3%) clinicians, respectively (Fig. 3D and E).

**Figure 4:**
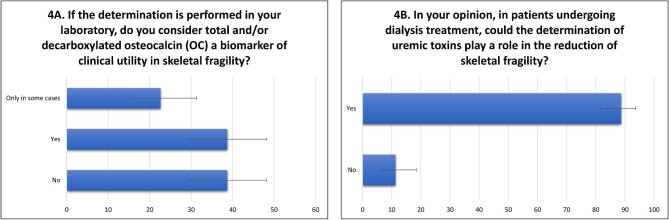
Utility of osteocalcin and uremic toxins in the management of skeletal fragility.

Concerning OC and uremic toxins in skeletal fragility, one third of clinicians (*n* = 41, 38.7%) consider OC a biomarker of clinical utility in skeletal fragility (*n* = 41, 38.7%) (Table S3, see online supplementary material). Only 24 participants (22.6%) declared that they found osteocalcin clinically useful and only in some selected cases (Fig. 4A). The utility of the determination of uremic toxins, instead, is widely recognized: 94 (88.7%) nephrologists consider it useful in the management of patients with skeletal fragility, and only 12 (11.3%) do not (Fig. 4B; Table S3, see online supplementary material).

### Management of BTMs in clinical practice, according to guidelines

**Figure 5:**
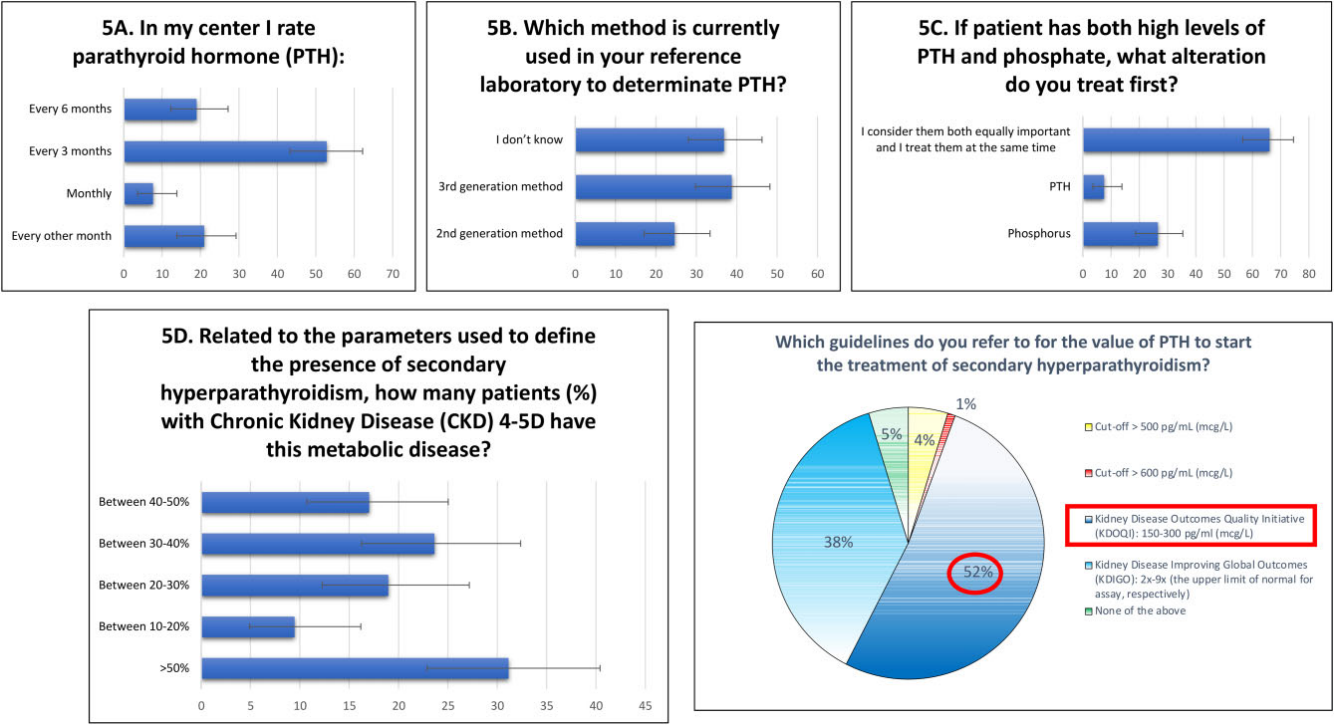
Use of PTH and management of sHPT.

As for the second issue (Table S4, see online supplementary material), most participants measure PTH levels: 56 (52.8%) every 3 months (Fig. 5A and B) and 20 (18.9%) every 6 months. High levels of PTH and phosphate were treated simultaneously by 70 (66%) participants (Fig. 5C). Regarding sHPT, 33 clinicians (31.1%) observed disease development in more than 50% of patients with CKD stage 4–5D (Fig. 5D). To start treatment of sHPT, the majority of clinicians use KDOQI guidelines (*n* = 55, 51.9%) whereas only 40 nephrologists (37.7%) relied on KDIGO guidelines which recommend referring to values of PTH between two and nine times the upper limit of normal range (Fig. 5E).

**Figure 6:**
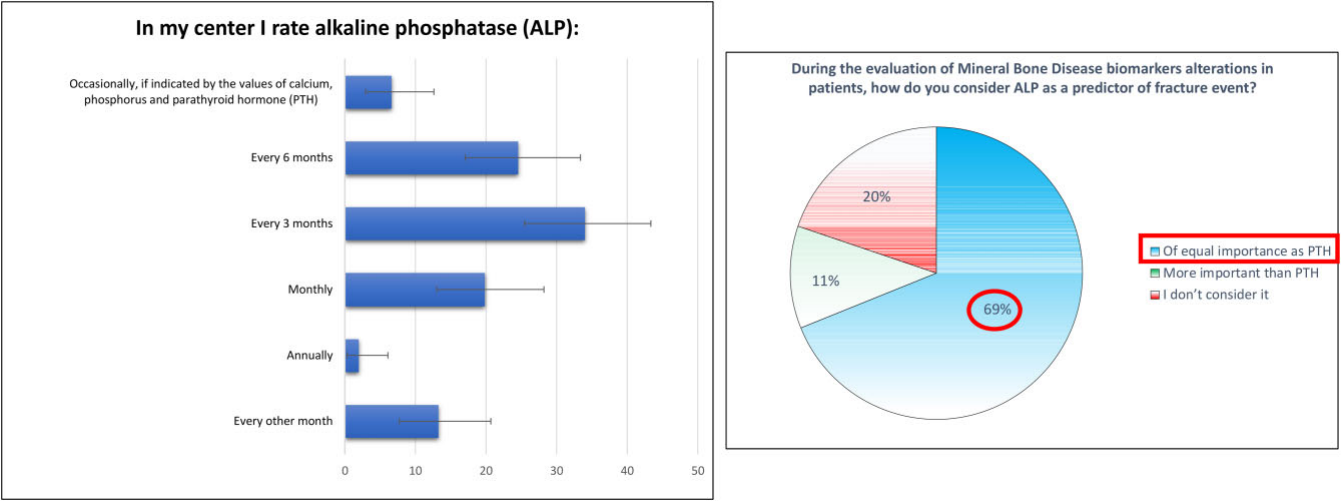
Use of alkaline phosphatase (ALP).

ALP was measured monthly by 21 clinicians (19.8%), and every 3 and 6 months by 36 (34%) and 26 (24.5%) clinicians, respectively (Fig. 6; Table S5, see online supplementary material). Most clinicians (*n* = 73, 68.9%) consider alterations of ALP of equal importance as alterations of PTH during the evaluation of CKD-MBD (Fig. 6).

## DISCUSSION

Two central topics were investigated, among Italian nephrologists, in this survey: (i) how BTMs are applied in clinical practice and (ii) whether nephrologists refer to guidelines to use them.

### Use of BTMs in clinical practice

Although bone biopsy remains the gold standard to know the correct bone remodeling in order to prevent fragility fractures, several recent studies have suggested that application of certain BTMs in clinical practice is extremely useful in defining bone turnover, assessing fracture risk and monitoring CKD-MBD therapy. Few prospective studies on the fracture event in CKD patients have shown that the best predictor for bone fracture is ALP [18]. Maruyama *et al*., analysing data from the Japanese dialysis registry, showed that in 185 277 patients, ALP levels were independently associated with mortality and hip fracture incidence [25]. Similarly, Iimori *et al*. demonstrated, in a single-center cohort study of 485 dialysis patients, that bALP was a useful marker for predicting the risk of fracture of any type [area under curve (AUC) = 0.766, *P* < .0001] [26].

Apart from ALP, other BTMs play a pivotal role in predicting fracture risk. Ueda *et al*. showed how serum P1NP values in hemodialysis patients, similarly to those of other biomarkers, correlated negatively with BMD values in the distal third of the radius: subjects with higher serum P1NP values had greater bone loss [27]. Furthermore, Okuno *et al*. confirmed the hypothesis that the serum values of CTX can provide important information on the degree of bone loss; in particular, evaluating the distal third of the radius in males in hemodialysis treatment, it was seen that increased CTX levels were associated with bone loss [28]. Finally, Shidara *et al*. found a significant association between the increase in serum TRAP-5b values and loss of cortical bone mass [29].

Several studies have used BTMs to assess cut-offs that may delineate high and low turnover. Sprague *et al*., analysing 492 dialysis patients, suggest that iPTH cut-offs to determine low and high bone turnover were <103.8 pg/ml (AUROC of 0.701) and >323 pg/ml (AUROC of 0.724), respectively [30]. Similarly Salam *et al*., in 69 patients with CKD stage 4–5 and on dialysis, defined the optimal PTH cut-off to define high turnover in five times the upper limit of normal [31]. Both studies underlined that bALP is better at discriminating low turnover than PTH, with an AUROC of 0.824 [31] and an AUROC of 0.757 if levels are < 33.1 U/L [30]. Furthermore, Jørgensen *et al*., in a retrospective cross-sectional study, showed that all BTMs were able to discriminate high and low turnover (AUROC >0.80), slightly lower for PTH and ALP (AUROC >0.75), P1NP >120.7 ng/ml was better at predicting high turnover (AUROC 0.88) and TRAP-5b <3.44 U/l at predicting low turnover (AUROC 0.82) compared to other BTMs. Moreover, the best performances were obtained through combinations of BTMs and, specifically for the high turnover, the combination between P1NP and TRAP-5b (AUROC 0.84, accuracy 90%) and for the low turnover the combination of bALP and TRAP-5b (AUROC 0.86, 78% accuracy) [32]. Likewise, Salam *et al*. who instead underlined that the best BTMs to define low turnover, in addition to bALP, were P1NP (AUROC 0.794) and TRAP-5b (AUROC 0.799) (Table 3) [31].

**Table 3:** Cut-off of BTMs values for the definition of high and low bone turnover according to Salam [31] and Jørgensen [32].

	High turnover		Low turnover	
	Salam	Jørgensen	Salam	Jørgensen
bALP, ug/L	>31	>33.7	<21	<24.2
Intact P1NP, ng/mL	>107	>120.7	<57	<49.8
TRAP-5b, U/L	>4.6	>5.05	<4.6	<3.44

In fact, the first work providing cutoff values for BALP for the discrimination between low and high bone turnover in dialysis patients had already been pointed out in 1996 by Ureña *et al*. [33].

In addition to thesek other BTMs such as CTX-I have been evaluated; its serum levels correlate significantly with histomorphometric measures of bone resorption and CTX is a predictive biomarker of high bone turnover (specificity 96%; positive predictive value 90%) [31]. Both Salam and Ginsberg underline the importance of this marker as an expression of high bone turnover [6, 31].

This Italian survey shows that, although 98.1% of nephrologists indicate that their reference laboratory satisfies the request for BTMs, most of the laboratories do not perform dosage of TRAP-5b, P1NP, and CTX. Furthermore, these BTMs defined as fundamental by studies described above are rarely used in clinical practice.

Regarding the Vitamin D-FGF-23 axis, the HOST study (randomized and double blind on 1099 patients with CKD G4-G5) showed that plasmatic values of 25(OH)D were correlated with those of 1.25(OH)_2_D (r = 0.43) and iPTH (r = 0.25) and as after a 2.9-year follow-up patients who were in lowest tertile of 1.25(OH)_2_D values had an increased risk of death (HR, 1.33; 95% CI, 1.01–1.74) and initiation of chronic dialysis (HR, 1.78; 95% CI, 1.40–2.26) [34]. Similarly, Scialla *et al*., in a cohort of 3860 patients with CKD stage 2–4 from the CRIC study, demonstrated that elevated FGF-23 was independently associated with an increased risk of cardiovascular events [35]. Recently Dörr *et al*., in a single-blind randomized trial, evaluated the effect of etecalcetide and alfacalcidiol therapy on progression of left ventricular hypertrophy (LVH) in 62 hemodialysis patients. The etelcalcetide-treated group, despite having much higher FGF-23 values at baseline, experienced a reduction in FGF-23 with a strong positive association with reduced left ventricular mass at 12 months compared with the alfacalcidiol-treated patients. PTH, phosphate, and αKlotho values were similar in the two groups, suggesting that FGF-23 as well as a marker of bone metabolism can be considered a marker of cardiovascular damage [36].

Our study population pays enough attention to the dosage of 25(OH)D by performing it every six months in 47.2% of cases; instead, in 63.2% of cases the determination of FGF-23 is never considered.

Osteocalcin (OC), a member of family of vitamin-K dependent proteins, plays a fundamental role in synthesis and regulation of the bone matrix by allowing the interaction between its Gla residues with calcium ions of hydroxyapatite [37]; it is present more in cortical bone than in trabecular bone and is a key element in bone strength [38]. OC, in normal conditions, could limit bone formation without causing demineralization of the bone. In conditions of OC deficiency, the onset of hyperostosis phenomena was observed: an increased osteoblastic surface, with greater deposition of bone matrix, has been seen in knock-out mice for OG1 and OG2, genes encoding for OC synthesis [39].

The VIKI study highlighted that patients with total OC values <150 μg/L had a threefold higher odds ratio of vertebral fractures than those with values ≥150 μg/L (OR = 3.15, 95% CI 1, 46–6.76, *P* = .003) [40]. Furthermore, significantly low OC values were found in patients with vascular calcifications [20].

In our survey, one third of clinicians (41, 38.7%) consider OC a useful biomarker in evaluation of skeletal fragility.

Conversely, most clinicians (88.7%) consider the determination of uremic toxins helpful in evaluation of skeletal fragility. In fact, the accumulation of uremic toxins during CKD favors the progression of bone disease across two pathways: skeletal resistance to PTH through the down regulation of the PTH1R receptor expressed by osteoblasts (mediated by indoxyl sulfate) and osteoblast dysfunction (mediated by p-cresyl sulfate or pCS) [41]. Uremic toxins, additionally, generate excess oxidative stress; some studies on mice have shown how advanced glycation end-products (AGE) modify the cross-links of type I collagen making bone less elastic and at greater risk of fracture [42, 43]. Other studies on bone quality impairment in rats with CKD showed that femoral bone elasticity inversely correlated with creatinine clearance, suggesting that CKD and increased uremic toxins are closely related to loss of bone quality [44]. These alterations determine the condition of ‘uremic osteoporosis’ different from primary osteoporosis found in the general population [41]. In fact, the difference between primary and secondary osteoporosis is relevant when considering fracture risk in CKD patients. The morphology of trabeculae, as well as the structure of cortical bone, are quite different in primary and uremic osteoporosis. Intense bone resorption by many multinucleated osteoclasts, induced by secondary hyperparathyroidism, is characteristic of high turnover hyperparathyroid bone disease.

### Management of BTMs in clinical practice in according to guidelines

The 2017 KDIGO CKD-MBD Guidelines recommend monitoring of phosphate, calcium, and PTH with a variable temporal frequency based on severity of abnormalities and degree of CKD progression. Calcium and phosphate should be monitored every 6–12 months in CKD G3a-G3b, every 3–6 months in CKD G4, and every 1–3 months in the G5 stage. PTH should be examined based on baseline and CKD progression in stage G3, every 6–12 months in stage G4, and every 3–6 months in stage G5 (including G5D). Although the ideal PTH value in non-dialysis patients is not known, it is recommended to maintain it between two and nine times the upper limit of normal in patients with CKD-G5D. Alkaline phosphatase should be monitored every 12 months in CKD G4-G5D (more frequently in case of elevated PTH). It is also recommended to measure markers of bone turnover, although not routinely [21]. The KDOQI working group, commented on the KDIGO guidelines, underlining how these are, in some points, conflicting or difficult to apply. Furthermore, the KDOQI groups reiterates the indication of maintaining PTH values between 150 and 300 pg/ml in patients with CKD-5D [45]. Recently, ‘European Consensus Statement on the Diagnosis and Management of Osteoporosis in Chronic Kidney Disease Stages G4-G5D’ was published and authors suggest monitoring BTMs for making diagnosis and monitoring treatment in patients with CKD, particularly using non kidney related BTMs such as bALP, P1NP, and TRAP-5b. In fact, these can provide important information after the start of treatment, given their rapid change, and if they are not suppressed after 3–6 months of antiresorptive therapy, it is necessary to evaluate adherence or presence of issue with drug used. Furthermore, these can be used to monitor patients who have discontinued treatment in order to detect loss of therapy effect and resumption of BMD reduction [13].

From this survey it emerges that, regarding PTH values, Italian nephrologists refer more to the KDOQI guidelines than to KDIGO (51.9% vs 37.7%) probably due to the clearer indication. Importantly, these ranges were obtained from bone turnover in cancellous bone, but it appears to be quite different in cortical bone. Future publications and research regarding bone metabolism, including the osteocyte role, might help in better defining a more reliable range of PTH levels in CKD patients.

Moreover, it emerges that PTH and alkaline phosphatase are monitored much more frequently than recommended by guidelines as they are considered of equal importance in the assessment of CKD-MBD and fracture risk. Concerning the treatment of sHPT, 66% of clinicians treat simultaneously high levels of phosphate and PTH, considering them of equal importance. Analysis of Dialysis Outcomes and Practice Patterns Study (DOPPS) data from USA and Canada shows how the parameter most frequently over the target is phosphate [46].

This study has several limitations. It was not possible to gather information on the nephrologists who took part in the survey, such as their geographic origin, the distribution between hub and spike nephrology departments, and university versus non-academic centers. Another limitation is the answer rate of only 10% of the target population of Italian nephrologists. The reason for the low answer rate can only be speculated. Being an online survey solicited by email, the low turnover is probably related to lack of time and to the overwhelming number of emails that all the doctors are receiving every day. Thus, there might be a selection bias and the nephrologists who answered the survey might be more interested in the topic of CKD-MBD. If this is true, the actual real-life picture regarding the use of PTH and BTMs in clinical practice might be worse than that represented by this study.

In conclusion, our results demonstrate how the diagnosis of CKD-MBD and the use of BTMs are extremely heterogeneous and how current guidelines do not give clear indications on their application. We need studies with clinical outcomes such as bone fractures and cardiovascular diseases in order to find the surrogate marker that best predicts them.

## Supplementary Material

sfad290_Supplemental_File

## Data Availability

The data underlying this article are available in the article and in its online supplementary material.
