# Supplementary material for: Real-world usage of Chronic Kidney Disease – Mineral Bone Disorder (CKD–MBD) biomarkers in nephrology practices
Source: Clin Kidney J. 2023 Nov 29;17(1):sfad290. doi: 10.1093/ckj/sfad290 (PMC10784916; doi:10.1093/ckj/sfad290)
Supplement: sfad290_Supplemental_File [file sfad290_supplemental_file.pdf]

SUPPLEMENTARY MATERIAL

Supplementary Table S1. Can the reference laboratory of your center fully satisfy all biomarkers requests that patients need in relation to the diagnosis and monitoring of MBD?

Ionized Calcium	Frequency	Frequency (%)	CI (95%)
No	2	1.9	0.32-6.09
Yes	104	98.1	93.91-99.68
Total	106	100	
**PTH**	**Frequency**	**Frequency (%)**	**CI (95%)**
No	1	0.9	0.048-4.56
Yes	105	99.1	95.44-99.95
Total	106	100	
**Alkaline phosphatase (ALP)**	**Frequency**	**Frequency (%)**	**CI (95%)**
No	1	0.9	0.048-4.56
Yes	105	99.1	95.44-99.95
Total	106	100	
**Vitamin K**	**Frequency**	**Frequency (%)**	**CI (95%)**
No	74	69.8	60.58-77.97
Yes	32	30.2	22.03-39.42
Total	106	100	
**Osteocalcin**	**Frequency**	**Frequency (%)**	**CI (95%)**
No	61	57.5	47,99-66,69
Yes	45	42.5	33,31-52,01
Total	106	100	
**Matrix Gla Protein (MGP)**	**Frequency**	**Frequency (%)**	**CI (95%)**
No	99	93.4	87.38-97.07
Yes	7	6.6	2.93-12.62
Total	106	100	
**N-terminal propeptide concentration of type I Procollagen (P1NP)**	**Frequency**	**Frequency (%)**	**CI (95%)**
No	94	88.7	81.55-93.72
Yes	12	11.3	6.28-18.45
Total	106	100	
**C-terminal telopeptide of type 1 collagen (CTX)**	**Frequency**	**Frequency (%)**	**CI (95%)**
No	76	71.7	62.57-79.65
Yes	30	28.3	20.35-37.43
Total	106	100	
**25-OH-vitamin D**	**Frequency**	**Frequency (%)**	**CI (95%)**
No	6	5.7	2.33-11.40
Yes	100	94.3	88.6-97.67
Total	106	100	
**1,25(OH)$_2$vitamin D**	**Frequency**	**Frequency (%)**	**CI (95%)**
No	45	42.5	33.31-52.01
Yes	61	57.5	47.99-66.69
Total	106	100	
**FGF-23 (intact molecule)**	**Frequency**	**Frequency (%)**	**CI (95%)**
No	94	88.7	81.55-93.72
Yes	12	11.3	6.28-18.45

Total	106	100	
**FGF-23 (c-terminal fragment)**	**Frequency**	**Frequency (%)**	**CI (95%)**
No	99	93.4	87.38-97.07
Yes	7	6.6	2.39-12.62
Total	106	100	
**Klotho (soluble)**	**Frequency**	**Frequency (%)**	**CI (95%)**
No	103	97.2	2.49-99.27
Yes	3	2.8	0.73-7.51
Total	106	100	
**Tartrate resistant-acid phosphatase 5b isoenzyme (TRAP5b)**	**Frequency**	**Frequency (%)**	**CI (95%)**
No	98	92.5	85.17-96.43
Yes	8	7.5	3.57-13.83
Total	106	100	

Supplementary Table S2. Use of other biomarkers.

Question	Answer	Frequency	Frequency (%)	CI (95%)
In my center I rate 25(OH)D:	Every other month	7	6.6	2.93-12.62
	Annually	4	3.8	1.21-8.85
	Every 3 months	35	33	24.57-42.38
	Every 6 months	50	47.2	37.81-56.69
	According to the values of calcium, phosphate and PTH	10	9.4	4.89-16.17
	Total	106	100	
**Question**	**Answer**	**Frequency**	**Frequency (%)**	**CI (95%)**
When do you consider the determination of FGF-23 and Klotho in patients with CKD-MBD?	Never	67	63.2	53.73-71.98
	Only in some cases	12	11.3	6.28-18.45
	Always to monitor the patient	27	25.5	17.87-34.40
	Total	106	100	
**Question**	**Answer**	**Frequency**	**Frequency (%)**	**CI (95%)**
If the determination is performed in your laboratory, do you refer to P1NP in your patients?	Up to stage CKD3A	2	1.9	0.317-6.09
	Up to stage CKD3B	7	6.6	2.93-12.62
	Up to stage CKD4-5D	14	13.2	7.72-20.68
	In every stage	27	25.5	17.87-34.40

	Never	56	52.8	43.31-62.19
	Total	106	100	
**Question**	**Answer**	**Frequency**	**Frequency (%)**	**CI (95%)**
If the determination is performed in your laboratory, do you refer to CTX in your patients?	Up to stage CKD3A	7	6.6	2.93-12.62
	Up to stage CKD3B	7	6.6	2.93-12.62
	Up to stage CKD4-5D	15	14.2	8.46-21.78
	In every stage	24	22.6	15.43-31.33
	Never	53	50	40.55-59.45
	Total	106	100	
**Question**	**Answer**	**Frequency**	**Frequency (%)**	**CI (95%)**
If the determination is performed in your laboratory, do you refer to TRAP-5b in your patients?	Up to stage CKD3A	2	1.9	0.317-6.09
	Up to stage CKD3B	4	3.8	1.21-8.85
	Up to stage CKD4-5D	13	12.3	7.00-20.06
	In every stage	26	24.5	17.05-33.38
	Never	61	57.5	47.99-66.69
	Total	106	100	

Supplementary Table S3. Utility of osteocalcin and uremic toxins in the management of skeletal fragility.

Question	Answer	Frequency	Frequency (%)	CI (95%)
If the determination is performed in your laboratory, do you consider total and/or decarboxylated OC a biomarker of clinical utility in skeletal fragility?	No	41	38.7	29.77-48.2
	Yes	41	38.7	29.77-48.2
	Only in some cases	24	22.6	15.43-31.33
	Total	106	100	
**Question**	**Answer**	**Frequency**	**Frequency (%)**	**CI (95%)**
In your opinion, in patients undergoing dialysis treatment, could the determination of uremic toxins play a role in the reduction of skeletal fragility?	No	12	11.3	6.28-18.45
	Yes	94	88.7	81.55-93.72
	Total	106	100	

Supplementary Table S4. Use of PTH and management of sHPT.

Question	Answer	Frequency	Frequency (%)	CI (95%)
In my center I rate PTH:	Every other month	22	20.8	13.83-29.25
	Monthly	8	7.5	3.57-13.83
	Every 3 months	56	52.8	43.31-62.19
	Every 6 months	20	18.9	12.26-27.16
	Total	106	100	
**Question**	**Answer**	**Frequency**	**Frequency (%)**	**CI (95%)**
Which method is currently used in the reference laboratory of your center to determinate PTH?	2nd generation method	26	24.5	17.05-33.38
	3rd generation method	41	38.7	29.77-48.2
	I don't know	39	36.8	28.02-46.27
	Total	106	100	
**Question**	**Answer**	**Frequency**	**Frequency (%)**	**CI (95%)**
In case the patient has high levels of PTH and phosphate, which of alterations do you prioritize in terms of treatment?	Phosphorus	28	26.4	18.69-35.41
	PTH	8	7.5	3.57-13.83
	I consider them both equally important and I treat them at the same time	70	66	56.64-74.57
	Total	106	100	
**Question**	**Answer**	**Frequency**	**Frequency (%)**	**CI (95%)**
Related to the parameters used to define the presence of sHPT, how many patients (%) with CKD stage 4-5D have this metabolic disease?	>50%	33	31.1	22.87-40.41
	Between 10-20%	10	9.4	4.89-16.17
	Between 20-30%	20	18.9	12.26-27.16
	Between 30-40%	25	23.6	16.24-32.36
	Between 40-50%	18	17	10.72-25.03
	Total	106	100	
**Question**	**Answer**	**Frequency**	**Frequency (%)**	**CI (95%)**
	Cut-off > 500 pg/mL (mcg/L)	5	4.7	1.75-10.14
	Cut-off > 600 pg/mL (mcg/L)	1	0.9	0.048-4.56
	Kidney Disease Outcomes Quality	55	51.9	42.39-61.28

Which guidelines do you refer to for the value of PTH to start the treatment of sHPT?	Initiative (KDOQI): 150-300 pg/ml (mcg/L)			
	Kidney Disease Improving Global Outcomes (KDIGO): 2x-9x (the upper limit of normal for assay)	40	37.7	28.89-47.24
	None of the above	5	4.7	1.75-10.14
	Total	106	100	

Supplementary Table S5. Use of alkaline phosphatase (ALP).

Question	Answer	Frequency	Frequency (%)	CI (95%)
In my center I rate ALP:	Every other month	14	13.2	7.72-20.68
	Annually	2	1.9	0.31-6.09
	Monthly	21	19.8	13.04-28.21
	Every 3 months	36	34	25.43-43.36
	Every 6 months	26	24.5	17.05-33.38
	Occasionally, if indicated by values of calcium, phosphate and PTH	7	6.6	2.93-12.62
	Total	106	100	
**Question**	**Answer**	**Frequency**	**Frequency (%)**	**CI (95%)**
During the evaluation of CKD-MBD, how do you consider ALP as a predictor of fracture event?	Of equal importance as PTH	73	68.9	59.59-77.13
	More important than PTH	12	11.3	6.28-18.45
	I don't consider it	21	19.8	13.04-28.21
	Total	106	100	
